# Aseptic Meningitis in Children: Analysis of 506 Cases

**DOI:** 10.1371/journal.pone.0000674

**Published:** 2007-08-01

**Authors:** Athanasios G. Michos, Vassiliki P. Syriopoulou, Christos Hadjichristodoulou, George L. Daikos, Evagelia Lagona, Panagiotis Douridas, Glykeria Mostrou, Maria Theodoridou

**Affiliations:** 1 First Department of Pediatrics, Aghia Sophia Children's Hospital, Athens University, Athens, Greece; 2 Department of Hygiene and Epidemiology, Medical Faculty, University of Thessaly, Larissa, Greece; 3 First Department of Propaedeutic Medicine, Laiko General Hospital, Athens University, Athens, Greece; Massachusetts General Hospital, United States of America

## Abstract

**Background:**

Non-polio human enteroviruses are the leading cause of aseptic meningitis in children. The role of enterovirus PCR for diagnosis and management of aseptic meningitis has not been fully explored.

**Methodology/Principal Findings:**

A retrospective study was conducted to determine the epidemiological, clinical, and laboratory characteristics of aseptic meningitis and to evaluate the role of enterovirus PCR for the diagnosis and management of this clinical entity. The medical records of children who had as discharge diagnosis aseptic or viral meningitis were reviewed. A total of 506 children, median age 5 years, were identified. The annual incidence rate was estimated to be 17/100,000 children less than 14 years of age. Most of the cases occurred during summer (38%) and autumn (24%). The dominant clinical symptoms were fever (98%), headache (94%) and vomiting (67%). Neck stiffness was noted in 60%, and irritation in 46% of the patients. The median number of CSF cell count was 201/mm^3^ with polymorphonuclear predominance (>50%) in 58.3% of the cases. Enterovirus RNA was detected in CSF in 47 of 96 (48.9%) children tested. Children with positive enterovirus PCR had shorter hospitalization stay as compared to children who had negative PCR or to children who were not tested (*P* = 0.01). There were no serious complications or deaths.

**Conclusions:**

Enteroviruses accounted for approximately one half of cases of aseptic meningitis. PCR may reduce the length of hospitalization and plays important role in the diagnosis and management of children with aseptic meningitis.

## Introduction

Aseptic meningitis refers to a clinical syndrome of meningeal inflammation in which common bacterial agents cannot be identified in the CSF [1]. Non-polio human enteroviruses (NPHEV) are the leading recognizable cause of aseptic meningitis accounting for 80% to 92% of all cases in which a pathogen is identified [1]–[3].

Enteroviruses constitute a genus of the picornavirus family which includes poliovirus types 1, 2, 3, and human enterovirus A, B, C, and D [4]. The NPHEV can cause a broad spectrum of illnesses such as febrile disease, hand-foot-mouth, herpangina, aseptic meningitis and encephalitis. Occasionally, NPHEV can cause severe infection with dismal outcome such as myocarditis and neonatal sepsis[1]–[3]. Most of the cases occur in epidemics during summer and autumn although sporadic cases can occur throughout the year [3], [5], [6]. In the USA alone NPHEV is estimated to cause 10 to 15 million symptomatic infections annually and at least 75,000 cases of aseptic meningitis [3].

The diagnosis of NPHEV infection is documented with viral cultures from tissue samples or cerebrospinal fluid (CSF). Among the limitations of viral culture for the diagnosis of NPHEV infection are a sensitivity of 65% to 75%, a turnaround time of 3 to 10 days, and the high degree of technical expertise required [1]. Serology also is of limited diagnostic value for enteroviral infections due to requirement to examine acute and convalescent serum samples for a large number of serotypes [3]. The polymerase chain reaction (PCR) has been shown to be an effective alternative to viral culture in diagnosing NPHEV meningitis and its use may reduce the hospital related cost by rapid diagnosis and earlier discharge from the hospital [3], [7]–[12]. In comparisons with viral cultures, PCR is more accurate with a sensitivity and specificity approaching 100% [8]–[11].

In this report we present the epidemiological, clinical, and laboratory characteristics of aseptic meningitis in our geographic region, and the role of Amplicor Eneterovirus PCR in the diagnosis and management of this disease.

## Materials and Methods

### Data collection

This retrospective study was conducted in the infectious diseases unit of “Aghia Sophia Children's Hospital” a tertiary care medical center serving approximately 70% of children with meningitis residing in the Athens metropolitan area. The medical records of children who were hospitalized from January 1994 to December 2002 and had as discharge diagnosis aseptic or viral meningitis were identified and reviewed. All children who had an acute illness with 10 or more white blood cells (WBC) in the CSF in the absence of microorganism on Gram stain and on routine culture, negative latex agglutination tests for bacterial antigens, and clinical course consistent with aseptic meningitis as well as those who had confirmed viral meningitis were included in the study. All children who had received antibiotics prior to CSF examination were excluded from the study. A questionnaire was used to collect information related to date of birth, gender, date of onset of illness, clinical presentation, laboratory findings in blood and CSF, PCR results when available, duration of antimicrobial therapy, days of hospitalization, and outcome. The study population included children 1 month to 14 years of age and was divided in 4 age groups: <1 year, 1–5 years (preschool), 6–12 years (elementary school), >12 years (secondary school). The study was approved by Aghia Sophia Children's Hospital review board and due to the retrospective nature of the study informed consents were waived.

PCR had been performed in 96 children by a commercially available reverse transcription based PCR (Amplicor Enterovirus test, Roche Diagnostics, Branchburg) according to the manufacturer's instructions. The decision for PCR testing was made by patients' attending physicians.

### Statistical analysis

The data were processed and analyzed by using the SPSS statistics software, version 12 for windows (SPSS, Inc.,Chicago,IL). Chi-square test was used to compare categorical variables and the Student́s *t-*test or non-parametric tests (Mann-Whitney *U* or Kruskal Wallis test) to compare continuous variables. Moreover, linear regression model was used to test for relation among the monthly mean temperature and viral meningitis hospitalizations. Annual incidence of aseptic meningitis was assessed by using as denominator the catchment population of the hospital. Population figures were based on the latest census available and predicted changes in the pediatric population in the Athens metropolitan area were taken into account. To investigate whether there was an association among the number of hospitalized cases each specific month and the mean monthly temperatures, data provided by the National weather forecast service for the years 1994–2002 were used.

## Results

### Epidemiology

**Figure 1:**
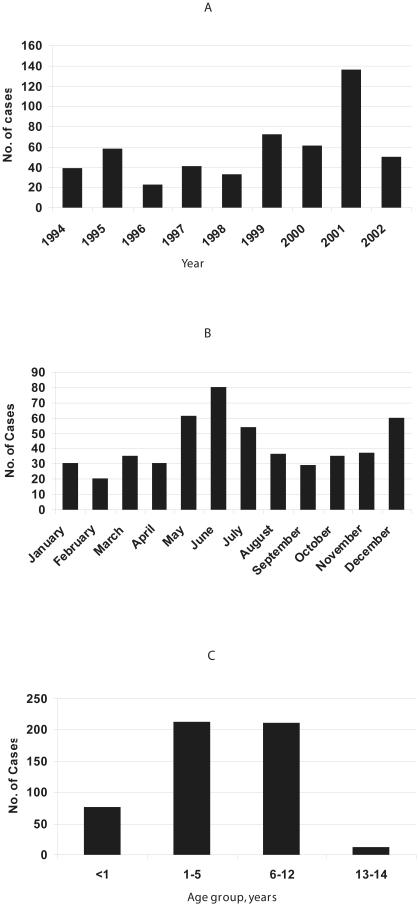
Distribution of Aseptic Meningitis Cases by Year (panel A), Month (panel B), and Age Group (panel C).

During the study period, 532 records of aseptic meningitis cases were identified of which 26 were excluded because of incomplete data or because they did not meet the enrollment criteria. A total of 506 patients were included in the analysis. The median age was 5 years (range, 1 month to 14 years), males outnumbered females by a ratio of 1.8 to 1. The mean annual incidence rate for the hospitalized aseptic meningitis cases in the region of Athens was estimated to be 17/100,000 children less than 14 years of age; the highest incidence was observed in the age group of 1–5 years (26/100,000 children) and the lowest in the age group of 13–14 years (7/100,000 in children). For infants <1 year old the incidence was 24/100,000 and for children 6–12 years of age the incidence was 14/100,000. The distribution of aseptic meningitis cases by year, month, and age group, of occurrence is shown in Figure 1. Although cases were observed throughout the year, most occurred during summer and autumn months; June to August, (38%) and September to November, (24%). The peak calendar month over the 9-year period was June with a total of 86 cases (17%). A larger number of cases was observed in the year 2001 as compared to preceding and following years, apparently related to community outbreaks having occurred this year as reported previously by Siafakas et al. [13]].

### Clinical, laboratory findings, and outcome

The patients admitted at the hospital within 32 hours (range 6h–96h) after the onset of symptoms. The dominant clinical symptoms upon admission were fever (98%), headache (94%), and vomiting (67%). Neck stiffness was noted in 60% of the patients, lethargy or irritation in 46%, and seizures in 2.3%. Other manifestations were anorexia (40%), rash (9%), symptoms of upper respiratory tract infection (4%), and diarrhea (1.6%). The mean duration of fever was 2.8 days (median, 1 day; range, 0–8 days) and the mean duration of hospitalization was 4.1 days (median, 4 days; range, 1.9– 8.5 days). Out of the 506 hospitalized children, 136 (26.9%) received antimicrobial therapy for more than 2 days, 311(61.5%) less than 2 days, and 59 (11.7%) did not receive any.

The initial blood laboratory values are summarized in Table 1. The WBC count was normal (<10,000/mm^3^) in 153 (30.2%) patients, slightly elevated (10–15,000/mm^3^) in 210 (41.5%) patients, and >15.000/mm^3^ in 143 (28.3%) patients. Notably, only 91 of 506 patients (18%) had lymphocytic predominance. CRP was normal in 47% of patients, mildly elevated (6–20 mg/dl) in 37,2%, and >21 mg/dl in 15.8% of children. Results of CSF examination are also presented in Table 1. Nineteen (3.8%) children had 10–25 cells/mm^3^, 91 (18%) children had 26–100 cells/mm^3^, 289 (57.1%) children had 101–500 cells/mm^3^, 75 (14.8%) children had 501–1000 cells/mm^3^ and 32(6.3%) children had more than 1000 cells/mm^3^. Lymphocytes predominance (>50%) was observed in 41.7 % of cases. The laboratory findings among the different age groups are shown in Table 2. No differences were observed among the groups with the exception of children <1 year old who had higher CSF protein and lower CSF glucose.

**Table 1:** Clinical Characteristics and Laboratory Findings for 506 Children with Aseptic Meningitis.

Age in years, median (IQR)	5 (3–8)
No. of males / No. of females	325/181
Duration of fever, hours, median (IQR)	24 (12–48)
Duration of hospitalization, days, median (IQR)	4 (3–6)
Antimicrobial therapy* >2 days, n (%)	136 (26.9%)
Antimicrobial therapy <2 days, n (%)	311(61.5%)
No antimicrobial therapy, n (%)	59 (11.7%)
WBC counts / mm^3^, median (IQR)	11,840 (9,500–15,200)
Lymphocytes >50 %, n (%)	91 (18 %)
Hemoglobin (mg/dl), median (IQR)	12 (11.4–12.8)
C-reactive protein, mg/dl, median (IQR)	7 (3–15)
**Cerebrospinal fluid**	
Cells/mm^3^ , median (IQR)	201 (117–417)
Lymphocytes >50%, n (%)	211 (41.7%)
Protein, mg/dL, median (IQR)	34 (21–53)
Glucose, mg/dL, median (IQR)	53 (47–60)

IQR = Interquartile range

* After lumbar puncture

**Table 2:** Laboratory Findings for 506 Children with Aseptic Meningitis by Age Group.

	Age groups, years				*P*
	<1	1–5	6–12	13–14	
	*n* = 73	*n* = 212	*n* = 208	*n* = 13	
White blood					
cells/mm3, median (IQR)	13,700 (9,900–17,300)	12,515 (9,900–15,720)	11,200 (9,000–14,100)	9,345 (7,900–11,000)	0.04
C-reactive protein, mg/dl , median (IQR)	8.5 (2–18.5)	8 (3–19)	5 (3–12)	12 (3–33)	NS
*Cerebrospinal fluid*					
cells / mm3 , median (IQR)	227 (144–547)	197 (112–382)	199.5 (110.5–402)	297 (114–467)	NS
glucose, mg/dl, median (IQR)	50 (44.5–59.5)	54 (47–60)	54 (48–62)	53(50–62)	0.02
protein, mg/dl, median (IQR)	44.5 (30–77)	28 (20–44)	36 (24–55)	40.5 (30–68)	<0.001
Enteroviral RNA, positive/tested (%)	4/8 (50)	21/46 (45.6)	22/41 (53.6)	0/1 (0)	NS

IQR = Interquartile range

The enterovirus PCR assay was performed in CSF samples of 96 patients. Enteroviral RNA was detected in forty seven (48.9%) of 96 children tested. Pertinent clinical and laboratory findings of PCR tested and not tested children are presented in Table 3. There were no significant differences among the PCR tested and not tested children with regard to age, gender, clinical features, and laboratory findings. Notably, children with positive enteroviral PCR had shorter hospitalization stay as compared to children who had negative PCR results and to children who were not PCR tested (*P* = 0.01). No fatalities, serious complications or sequelae occurred among the hospitalized patients.

**Table 3:** Comparison of Enterovirus PCR in Tested and not Tested Children

	Enterovirus PCR			
	positive	Negative	not tested	P
	*n* = 47	*n* = 49	*n* = 410	
Age, years, median (IQR)	5 (4–9)	5 (3–8)	5 (3–7)	0.4
No. males/No. females	27/20	33/16	265/145	0.57
Fever				
yes	46	49	398	0.46
no	1	0	12	
Headache				
yes	45	46	376	0.8
no	2	3	27	
Vomiting				
yes	31	35	263	0.65
no	16	13	133	
Seizures				
yes	1	0	10	0.53
no	44	47	375	
Neck stiffness				
yes	25	30	244	0.64
no	21	19	153	
Exanthema				
yes	3	5	34	0.79
no	44	44	375	
White blood cells/mm^3^, median (IQR)	12,500 (9,890–16,300)	11,600 (9,800–15,390)	11,800 (9,110–15,000)	0.59
Cerebrospinal fluid				
cells/mm^3^, median (IQR)	200(129–322)	195(107–347)	202(117–447)	0.7
glucose, mg/dl, median (IQR)	55(49–63)	53.5(47–62.5)	55(49–63)	0.34
protein, mg/dl, median (IQR)	32(20–63)	29(20–50)	35(21–53)	0.6
Days of hospitalization, median (IQR)	3 (2–5)	4 (3–6)	4 (3–5.5)	0.01
Days of antibiotic administration, Median (IQR)	2(1–3)	4(1–5)	3(2–5)	0.08

IQR = Interquartile range

## Discussion

Distinguishing aseptic from bacterial meningitis is not always easy due to considerable overlap in clinical symptoms and laboratory findings. Uncertainty in diagnosis results in prolonged hospitalization and unnecessary use of antibiotics. In the present study, it was shown that the use of enterovirus PCR in children with aseptic meningitis may shorten the hospital stay and decrease the use of antimicrobial agents.

The overall annual incidence of aseptic meningitis in our region was estimated to be 17 cases / 100,000 children less than 14 years of age. This rate is higher than that reported by other countries with the exception of those reported during epidemics [12]. Enteroviruses accounted for approximately one half of the cases where PCR was performed on CSF. In a recent study from Canada, enterovirus was detected by PCR in 54.3% of children with aseptic meningitis having similar clinical characteristics with our patients [7]. During the 9-year study period an enterovirus epidemic occurred in the year 2001 in Greece which resulted in doubling the number of hospitalized children with aseptic meningitis [13]. Similarly with previous reports [ 3], [5]–[8], most cases of aseptic meningitis occurred during summer throughout the study period, however, no association was found between the number of hospitalized aseptic meningitis cases and the mean monthly temperatures (data not shown). The incidence of aseptic meningitis depends more on the yearly outbreaks as well as the yearly trends.

Many times the diagnosis of viral meningitis is based only on clinical criteria and conventional laboratory methods. There are studies that have developed clinical prediction algorithms to help excluding the possibility for bacterial meningitis [14]. The standard laboratory tests most of the time are not specific, as peripheral WBC counts vary widely and occasionally during the initial stages of aseptic meningitis may show polymorphonuclear predominance. CSF examination also does not always help to distinguish aseptic from bacterial meningitis since the number of cells vary from few to thousands and early in the course of the disease, in some cases, there is polymorphonuclear predominance [12], [15], [16]. Moreover, highly elevated CSF white cell counts and low CSF glucose concentration may be observed in a minority of cases [12], [16]. In our population we found statistically significant increased protein and decreased glucose levels in children less that 1 year old. In view of the overlapping clinical and laboratory manifestations of bacterial and viral meningitis, occasionally in clinical practice our decisions may be wrong with serious consequences for the patients.

Until recently, the laboratory confirmation of viral meningitis was based upon isolation of the causative agent in cell cultures. While this method is useful, a high degree of technical skill is required and several days are needed before viral growth and identification. Thus, results are not available at the time when management decisions must be made [17]. Because of the large number of serologically distinct types of enteroviruses, serology also can not be used as a routine tool in the diagnosis of aseptic meningitis. Over the last decade, progress has been made in the diagnosis of enterovirus meningitis through molecular biology techniques. The use of PCR reduces the time required for identification of the causative agent and may decrease the use of empiric therapy with antibiotics and shorten hospitalizations.

Indeed, in agreement with previous observations [12], [18]–[22], enterovirus PCR reduced significantly the length of hospital stay in our medical center. In another study, although the time of reporting PCR results (less or more than 24 hours) had no significant reduction in antibiotic use or length of hospitalization, the earlier report of results reduced by 37% the hospital related expenses [23]. Therefore, rapid diagnosis of viral meningitis with PCR could have a large impact on hospital costs.

Several limitations of the present study should be pointed out. First, it is a retrospective study and the relevant information has been collected from patients' medical records. Second, assumptions about incidence of aseptic meningitis have been based on the patients presenting to the index hospital, whereas several children might go to other health care settings serving the same population. Therefore, the incidence of the disease is likely underestimated. Finally, the patients tested or not tested with enteroviral PCR were selected by physicians, based on their clinical judgment and not on predefined criteria. This way of selection might have affected the length of hospital stay.

Whether a clinical microbiology laboratory should add enterovirus PCR in the conventional strategy for the diagnosis of viral meningitis and at what frequency the assay should be performed during epidemic and not epidemic periods can not be answered by the present study. The optimal cost-benefit strategy for the management of aseptic meningitis needs to be determined in future studies by addressing these issues.
